# The Genomic Scrapheap Challenge; Extracting Relevant Data from Unmapped Whole Genome Sequencing Reads, Including Strain Specific Genomic Segments, in Rats

**DOI:** 10.1371/journal.pone.0160036

**Published:** 2016-08-08

**Authors:** Robin H. van der Weide, Marieke Simonis, Roel Hermsen, Pim Toonen, Edwin Cuppen, Joep de Ligt

**Affiliations:** 1 Hubrecht Institute, Royal Netherlands Academy of Arts and Sciences (KNAW), University Medical Centre Utrecht, Utrecht, The Netherlands; 2 Division of Gene Regulation, The Netherlands Cancer Institute, Amsterdam, The Netherlands; Xiamen University, CHINA

## Abstract

Unmapped next-generation sequencing reads are typically ignored while they contain biologically relevant information. We systematically analyzed unmapped reads from whole genome sequencing of 33 inbred rat strains. High quality reads were selected and enriched for biologically relevant sequences; similarity-based analysis revealed clustering similar to previously reported phylogenetic trees. Our results demonstrate that on average 20% of all unmapped reads harbor sequences that can be used to improve reference genomes and generate hypotheses on potential genotype-phenotype relationships. Analysis pipelines would benefit from incorporating the described methods and reference genomes would benefit from inclusion of the genomic segments obtained through these efforts.

## Background

Next-generation sequencing (NGS) is used in a large variety of applications ranging from single cell analyses to complex microbial communities and complete vertebrate and plant genome analyses [1]. NGS reads are, in general, aligned to an organism-specific reference genome as a first step in data analysis. Such reference genomes are typically derived from a single individual, animal or strain, with the exception of the human reference genome. Reads that align (map) to the reference genome are subsequently used for data analysis, while the unmapped reads are usually discarded [2,3]. Failure to map against the reference genome can be due to two mechanisms: 1) errors occurred in the sequencing process and as a consequence the read does not faithfully represent the original DNA fragment, or 2) the sequence captured in the read is not, or only partially, present in the reference assembly used for mapping. Filtering out reads originating from the first source is fairly straightforward and implemented in most data processing procedures by discarding reads with low quality scores [4,5]. The second source of unmapped reads often contains sequences from exogenous species due to experimental and sampling contamination [5–8]. Software has been developed that removes this class of sequencing reads [5,6,9,10], however most analysis pipelines do not include such a filtering step, resulting in lower mapping percentages or falsely mapped reads due to the greedy nature of NGS mapping algorithms [11]. Interestingly, a fair portion of unmapped reads remain when sequencing-artifacts and contaminations are removed [12]. With broad applicability of NGS methods, there is an increasing interest in the source of unmapped reads. Previous work has shown that there is relevant information to be discovered [8,13–16]. We set out to determine what type of sequences are present in this "scrapheap" of data obtained in the context of the genomic characterization of more than thirty widely used laboratory rat strains [*Rattus norvegicus and Rattus rattus*] [17,18].

High quality unmapped reads can contain biological information from loci that are missing from the species reference assembly including strain specific segments. Laboratory rat strains have diverged relatively recently but strain-specific genomic segments can be acquired through, for example, retroviral activity or lost in other strains through genetic drift [19,20]. While copy number variation and non-reference sequence variation have been shown to exist in mouse [*Mus Musculus*] strains and contribute to diversity [21,22], the sources and promises of unmapped reads have not been investigated in a systematic way. Several recent studies use similar approaches to extract biological relevant information from unmapped reads however these do not provide the underlying source code, making the methods hard to reproduce and implement [12,23].

The large amounts of inbred rat-(sub)strain complete genome sequences generated in the last decade provide a high quality dataset [24–27] which we used for development and implementation of a systematic un-mapped read analysis. While the rat reference genome has continuously been improved since its release in 2003 [28], to date, no strain specific segments have been included, in contrast to the most recent version of the human reference genome, GRCh38, which does include alternative loci for complex and highly variable regions [29].

## Results

**Fig 1:**
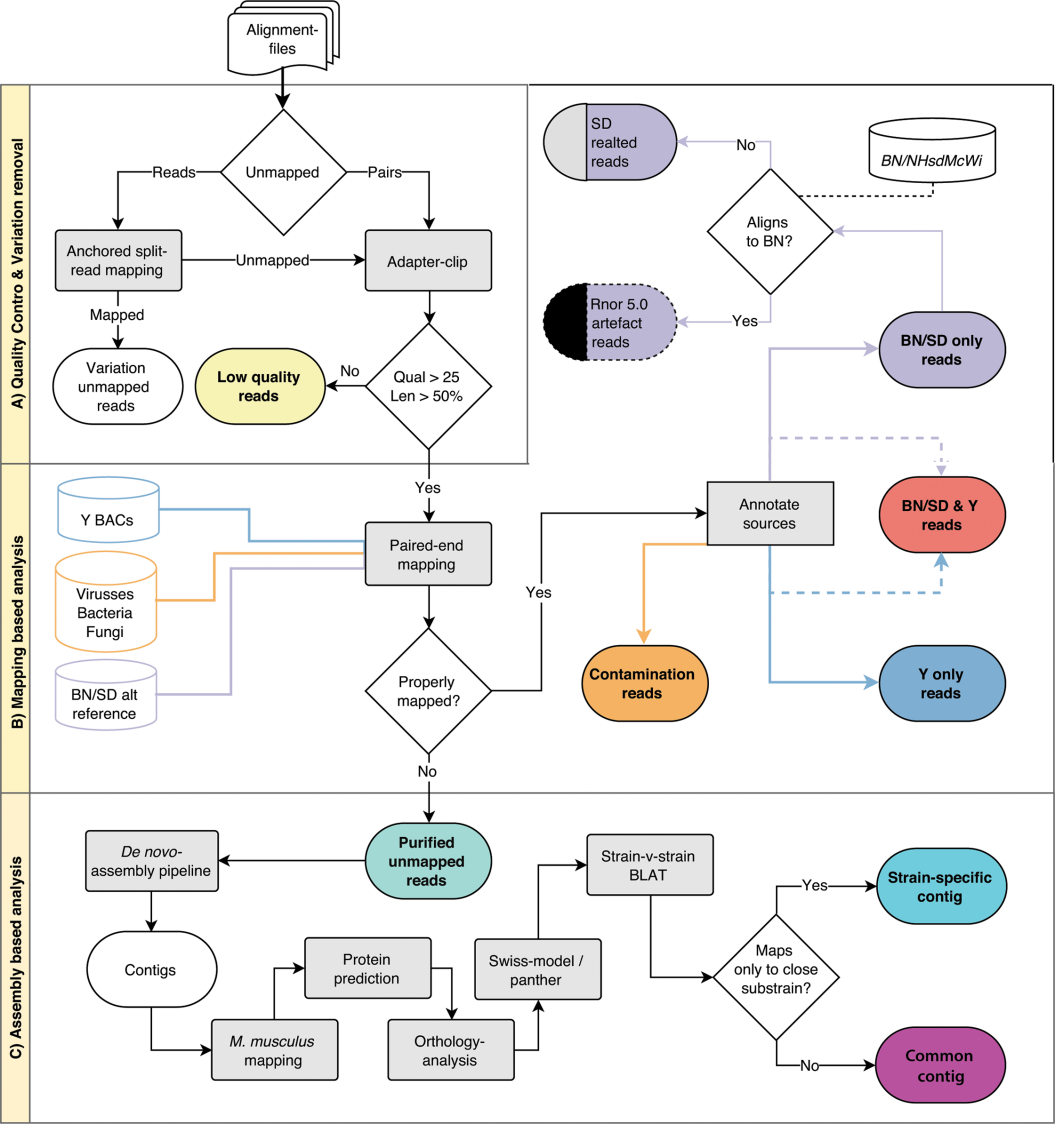
Filtering and processing workflow. Alignment files went through three stages; A) Quality Control, to remove low quality reads and reads affected by genomic variation, B) Mapping-based filtering, to identify reads derived from contaminants and regions present in alternative reference sequences and C) Identification of possible biological function and classification of common / strain-specific status.

**Fig 2:**
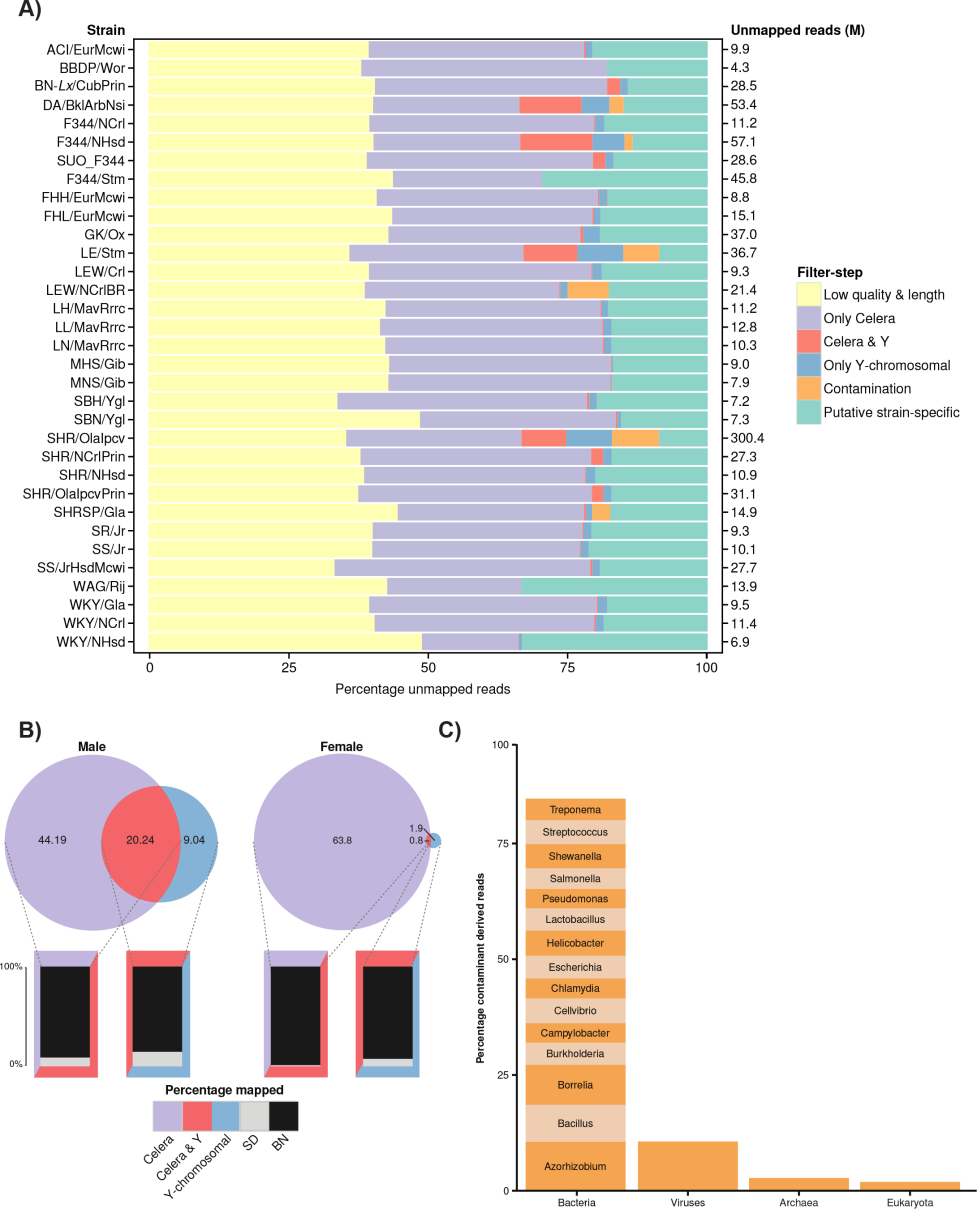
Origins of Unmapped reads. **A)** Stacked bar-graph of the origins of unmapped reads per strain. Total number of unmapped reads (millions) per strain is displayed on the right vertical axis. B) Distribution of reads, mapping to the alternative reference genome (Celera) and/or to the Y-chromosomal BAC-contigs of SHR/Akr, for male and female samples. C) Contaminant contribution in the twelve samples with contaminant-derived reads. The used contaminant-database consists of RefSeq-genomes of bacteria, viruses and fungi.

We aligned whole genome sequencing (WGS) data of 33 rat strains to the latest rat reference genome assembly (BN/NHsdMcWi, RGSC5.0) to identify ‘unmappable’ reads (Table 1). Large differences were observed in the absolute amounts of unmapped reads (between 2 and 150 million) per (sub)strain. The highest amount of unmapped reads was found for SHR/Olalpcv, possibly due to the older, less accurate, sequencing technology used (Illumina GAII). Unmapped reads were subjected to a series of filtering steps (Fig 1). In the first step, we filtered out reads with low base-call quality scores (phred <25) and low read-length (< = 50% of the expected read-length) as well as reads that were unmapped due to known genetic variation (Fig 1A). On average 40.5±3.5% of the unmapped reads were removed by these criteria (Fig 2A). We observed that the fraction of remaining reads was independent of sequencing platform or genomic coverage. Interestingly, more than half of the reads pass Quality Control (QC) criteria and likely represent true biological sequences.

**Table 1:** Study overview.

Sample	Published in	Low QC	Celera	Celera & Y	Y	Contami-nation	Remaining
ACI/EurMcwi	Atanur, S. et al. (2013)	39%	39%	0%	1%	0%	21%
BBDP/Wor	Atanur, S. et al. (2013)	38%	44%	0%	0%	0%	18%
BN-*Lx*/CubPrin	Hermsen, R. et al. (2015)	41%	42%	2%	1%	0%	14%
DA/BklArbNsi	Guo, X. et al. (2013)	40%	26%	11%	5%	3%	15%
F344/NCrl	Atanur, S. et al. (2013)	39%	40%	0%	2%	0%	18%
F344/NHsd	Guo, X. et al. (2013)	40%	26%	13%	6%	1%	13%
SUO_F344	Hermsen, R. et al. (2015)	39%	40%	2%	1%	0%	17%
F344/Stm #	Unpublished	44%	27%	0%	0%	0%	30%
FHH/EurMcwi	Atanur, S. et al. (2013)	41%	40%	0%	1%	0%	18%
FHL/EurMcwi	Atanur, S. et al. (2013)	44%	36%	0%	1%	0%	19%
GK/Ox	Atanur, S. et al. (2013)	43%	34%	1%	3%	0%	19%
LE/Stm	Atanur, S. et al. (2013)	36%	31%	10%	8%	6%	9%
LEW/Crl	Atanur, S. et al. (2013)	39%	40%	0%	2%	0%	19%
LEW/NCrlBR	Atanur, S. et al. (2013)	39%	35%	0%	1%	7%	18%
LH/MavRrrc	Atanur, S. et al. (2013)	42%	38%	0%	1%	0%	18%
LL/MavRrrc	Atanur, S. et al. (2013)	41%	40%	0%	1%	0%	17%
LN/MavRrrc	Atanur, S. et al. (2013)	42%	39%	0%	1%	0%	17%
MHS/Gib	Atanur, S. et al. (2013)	43%	40%	0%	0%	0%	17%
MNS/Gib	Atanur, S. et al. (2013)	43%	40%	0%	0%	0%	17%
SBH/Ygl	Atanur, S. et al. (2013)	34%	45%	0%	1%	0%	20%
SBN/Ygl	Atanur, S. et al. (2013)	49%	35%	0%	1%	0%	15%
SHR/OlaIpcv	Atanur, S. et al. (2010)	35%	31%	8%	8%	8%	9%
SHR/NCrlPrin	Hermsen, R. et al. (2015)	38%	41%	2%	1%	0%	17%
SHR/NHsd	Atanur, S. et al. (2013)	39%	40%	0%	2%	0%	20%
SHR/OlaIpcvPrin *	Hermsen, R. et al. (2015)	38%	42%	2%	1%	0%	17%
SHRSP/Gla	Atanur, S. et al. (2013)	45%	33%	0%	1%	3%	17%
SR/Jr	Atanur, S. et al. (2013)	40%	38%	0%	1%	0%	21%
SS/Jr	Atanur, S. et al. (2013)	40%	37%	0%	1%	0%	21%
SS/JrHsdMcwi	Atanur, S. et al. (2013)	33%	46%	0%	1%	0%	19%
WAG/Rij	Atanur, S. et al. (2013)	43%	24%	0%	0%	0%	33%
WKY/Gla	Atanur, S. et al. (2013)	39%	41%	0%	2%	0%	18%
WKY/NCrl	Atanur, S. et al. (2013)	40%	39%	0%	1%	0%	19%
WKY/NHsd	Atanur, S. et al. (2013)	49%	17%	0%	1%	0%	33%

Analyzed strains and the origins of unmapped reads in percentages

* Illumina GAII sequencing platform

# Mate-pair library

### Missing sequences in the reference genome

To avoid inclusion of reads that represent genuine reference genome information but could not be mapped due to reference genome gaps, we included raw sequencing reads from the original reference strain (BN/SsNHsdMCW) in our analysis and also utilized an alternative rat genome assembly that is based on two strains (BN/SsNHsdMCW & SD). We aligned the purified unmapped reads to an alternative rat genome assembly (generated by Celera) to identify unmapped reads due to an incomplete reference. The alternative genome assembly, referred to as ‘Celera’, is a hybrid consisting of 79% Brown Norway (BN/SsNHsdMCW) and 21% Sprague Dawley (SD) data [30]. The majority of the high-quality reads (62.1±14.2%) could be mapped to this assembly. To identify reads that aligned to Celera due to missing sequences in RGSC5.0, we annotated the Celera assembly with regions covered by WGS-data of BN/SsNHsdMCW [31]. The vast majority, 93.0±20.5%, of the unmapped reads mapped to these regions (Fig 2B), highlighting the incompleteness of the current RGSC5.0 assembly. The remaining reads that mapped to the Celera assembly (7%) could reflect strain-specific segments, shared with SD, which are lost/absent in BN/SsNHsdMCW.

### The Y-chromosome is a source of unmapped reads

Both RGSC5.0 and Celera were based on DNA from female animals and do not contain Y-chromosomal contributions. To determine the amount of unmapped reads due to this omission, purified reads were mapped to sequences of the recently described Y-chromosomal BAC-contigs of SHR/Akr [32]. Four strains showed significantly more reads mapping to these BAC’s: on average 6.7±1.6% of the total unmapped reads (P<0.01). Of those, two are known to be male (Da/BklArbNsi and F344/NHsd), while the other two are originally described as female samples (LE/Stm and SHR/Olalpcv). The latter could be a result from sequencing male animals, however the X-chromosomal coverage depth is similar to the autosomes, arguing against a male sample. Alternatively, these strains may have larger pseudo-Y-chromosomal segments present on the X-chromosome [33] (Fig 2B). Another 12% of the purified reads from these four strains map to both the Y-chromosomal BAC-contigs and to the alternative reference. Of these, 75.7±6.4% maps to sequences missing in the RGSC5.0 assembly, suggesting they could derive from homologous sequences in (pseudo) autosomal regions (Fig 2B).

### Contamination is not a constant factor

A possible source of high quality unmapped reads is contamination. We aligned the remaining unmapped reads to a contamination-database consisting of prokaryotic, viral and fungal RefSeq-genomes (V.61). Other likely contaminants (e.g. parasites, human and mouse) are not included, as their high sequence-homology with rat could lead to removal of rat material due to greedy mapping [11]. The identification of these possible *animalia*-derived contaminations is performed in the OrthoMCL-analysis later in this study.

Contamination-derived reads were found in 12 samples, with a contribution of more than 1% (median: 4.8%) in 6 experiments (Da/BklArbNsi, F344/NHsd, LE/Stm, LEW/NCrlBR, SHR/Olalpcv and SHRSP/Gla) (Fig 2A). Bacterial-derived reads were the largest contaminant-superkingdom with 88.4%, no bias was observed for a specific genus (Fig 2C). Apart from the positive control sample in Illumina machines -PhiX174- and *herpesviridae*, no laboratory-specific contaminants were identified.

A large fraction (~70%) of strain-specific contigs that show no homology to mouse consist of (proto)bacteria (Figure D in S1 File), suggesting a large contribution of feces-derived bacterial contamination. The three strains sequenced in East-Asia (F344/Stm, F344/NHsd and DA/BklArbNsi) had significantly higher amounts of predicted peptides with orthologs in the roundworm *Brugia malayi* (P<0.05). This roundworm is known to cause Brugian elephantiasis, a rare form of lymphedema, and lives in Eastern Asia [34]. This biologically relevant finding shows that our workflow allows identification of (contamination) sequences from members of the *Animala*-kingdom.

### Cross strain similarity of unmapped reads resembles phylogeny

**Fig 3:**
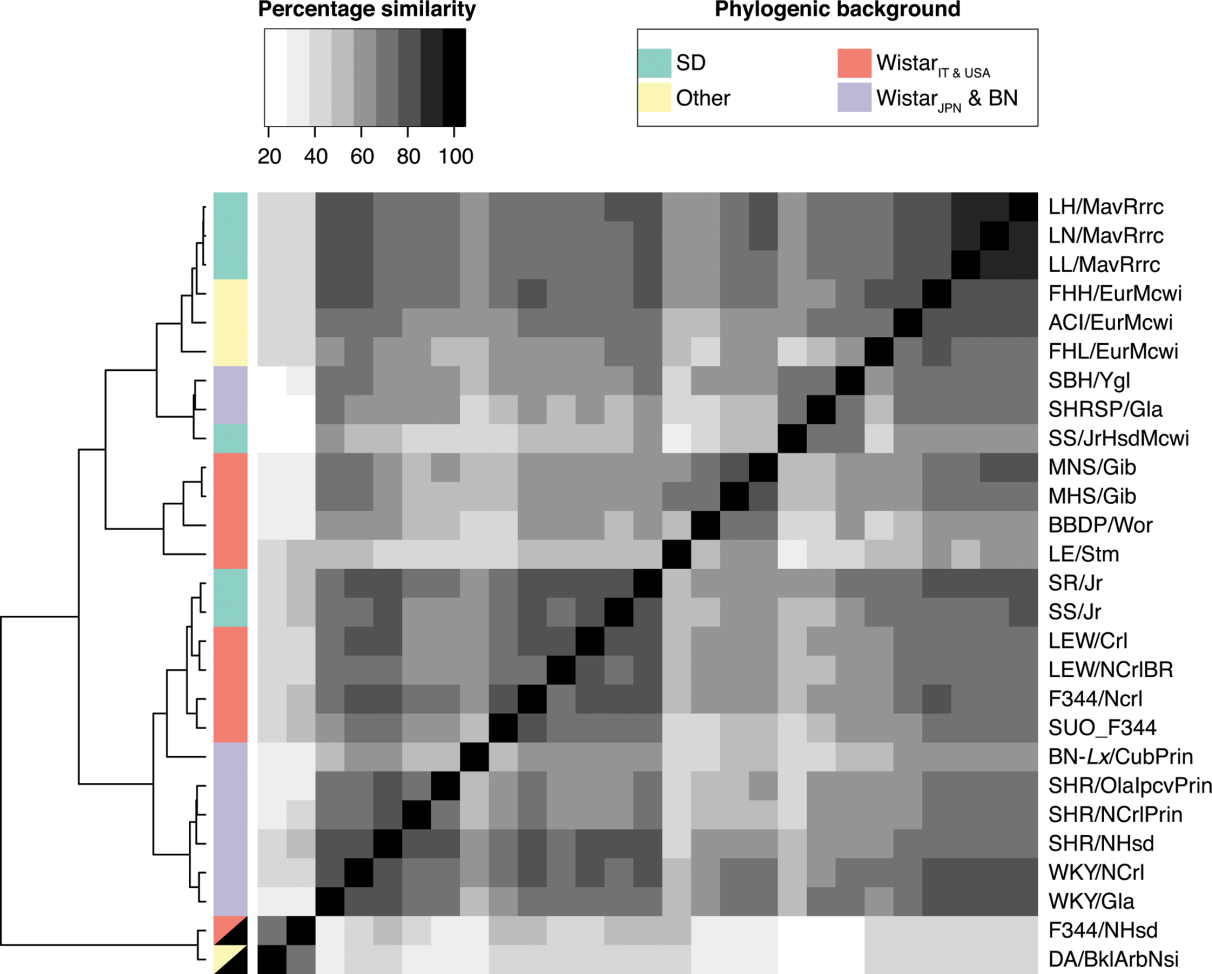
Similarity-clustering of unmapped reads. Black semi-filled phylogenic background denotes male samples. Euclidean distance-based clustering of strains on basis of the percentage between-strain read-pair sequence similarity, with a minimal sequence similarity of two non-overlapping blocks of 34bp.

Reads passing the previous filtering steps were most likely to contain strain specific sequences. To investigate the cross-strain similarity between the remaining reads, samples were clustered based on their between-strain sequence similarity. A large fraction (66%) of the reads had similar (>70% identity) sequences in more than one sample. The resulting similarity matrix shows a strong resemblance to previously established phylogenies of inbred rat strains [26,35,36] (Fig 3). An exception is the gender, which is reflected more strongly in the clustering than the phylogeny. This is likely due to the fact that the used Y-contigs are from a strain with a relatively short Y-chromosome. The used male samples can therefore still have a lot of overlap in Y-derived reads after filtering, simply because these have larger Y-chromosomes [37]. The phylogeny is reflected in sub-strains with a shared ancestor-strain, for example the Lyon Hypertensive strains (LH/LN/LL). Interestingly, more distant relationships are also reflected in the similarity matrix, as shown by the clustering of strains derived from the Italian colony of the outbred Wistar rat (MHS/Gib and MNS/Gib). The observation that unmapped reads follow known evolutionary patterns, suggests that strain-specific/non-reference genomic segments can harbor biological interesting strain-specific/non-reference genomic segments.

### *De novo* assembly

**Fig 4:**
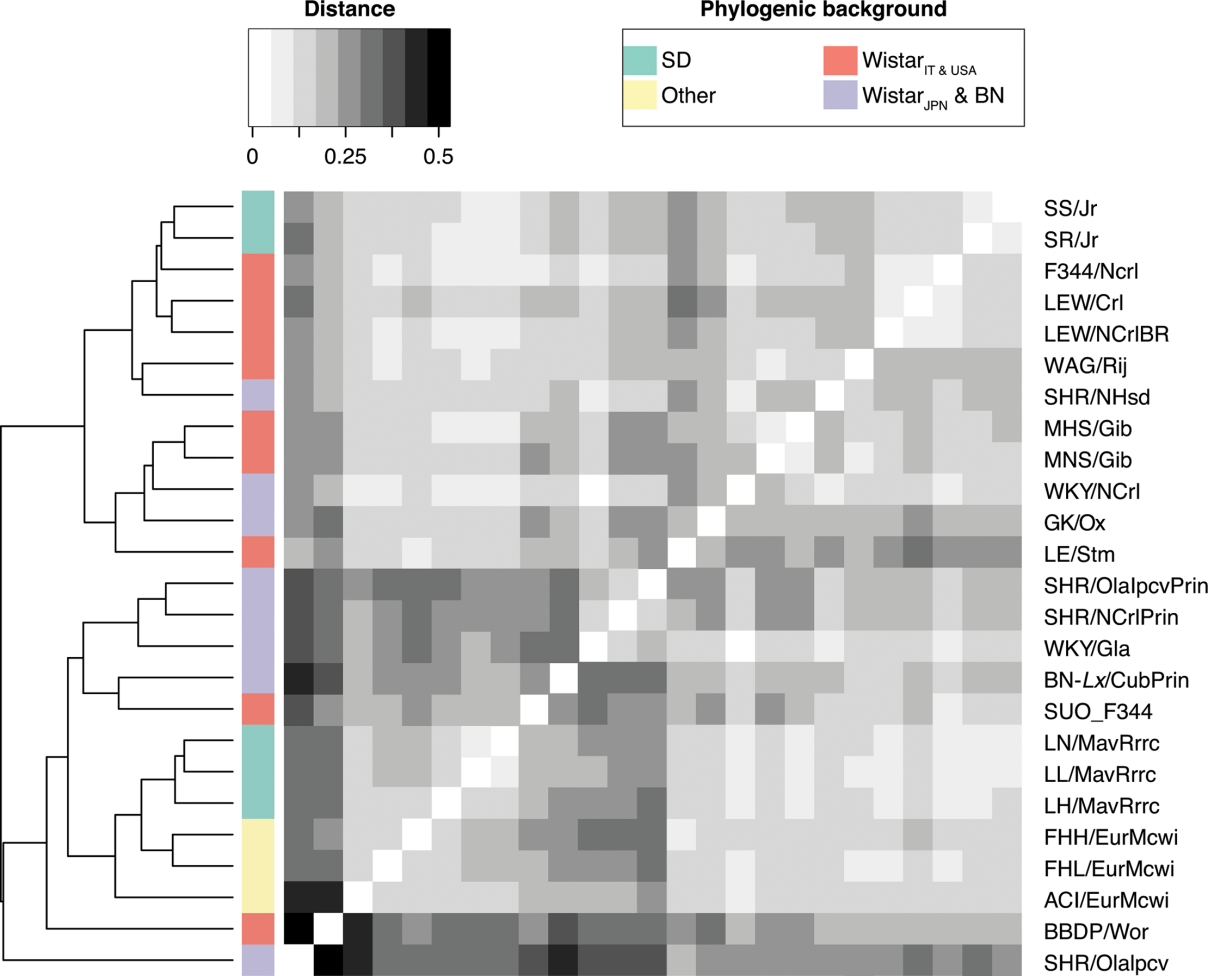
Distance-clustering of strain-assemblies. Euclidean distance-based clustering of strains on basis of the pairwise distances.

To assess the function and characteristics of these putative strain-specific segments, we assembled the reads used for the similarity clustering with a *de novo* assembly pipeline (SOAPdenovo-based, see methods), yielding on average 11Mb of assembled contigs per sample. The average weighted median of contig-sizes per sample (N50) was 910±121bp (Figure A in S1 File). Although repetitive DNA (like satellite DNA) can be a source of species diversity, these did not assemble to contigs, which is a known limitation of short read sequencing and *de novo* assembly methods [38]. Overall, the *de novo* assembly resulted in 94,759 contigs larger than 1Kb including 112 larger than 10Kb, in 30 strains. Distance clustering of substrain-assemblies showed strong resemblance to the phylogeny of *R*. *norvegicus* (Fig 4).

There were 3 samples that yielded a larger amount of contigs, as well as larger contigs: the males (Da/BklArbNsi and F344/NHsd) and F344/Stm, which were sequenced using mate-pair sequencing, a different library preparation approach with a larger insert size. Several other samples resulted in low-quality assemblies, in particular SBN/Ygl and SHR/NHsd. These 2 samples yielded no contigs larger than 1Kb and had an average N50 of 129bp. These samples had a relatively low sequencing coverage, although other well performing strains had a similar low coverage (Figure B in S1 File). These two samples do show a strong shift in GC-content between the total set of reads and the reads used for *de novo* assembly (Figure B in S1 File). Strong GC-bias (difference >2%) has been shown to aggravate *de novo* assembly due to unequal coverage in the genome [39].

### Identified sequences and peptides

To distinguish between sequences present in the majority of strains (presumably missing in the reference genome and/or SD) and those that are strain-specific, we aligned the *de novo* assembled contigs of all samples and denoted the contigs identified within a single strain to be strain-specific (Fig 1C). By performing a BLAT search for all contigs against the rat trace archives, [v105, ftp://ftp.ncbi.nlm.nih.gov/pub/TraceDB/], we found that 0.1% of the common contigs showed a reciprocal overlap of 66% or more with rat trace archives (0.0028% with ≥85% or more), indicating that only a very small portion overlaps or extends existing archive sequences while the majority are novel sequences and are unlikely to represent gaps in the current rat reference genome. For the contigs that were classified as strain-specific, this was 0.02% (0.0018% with ≥85% overlap), showing an even greater enrichment for novel sequences, as expected.

**Fig 5:**
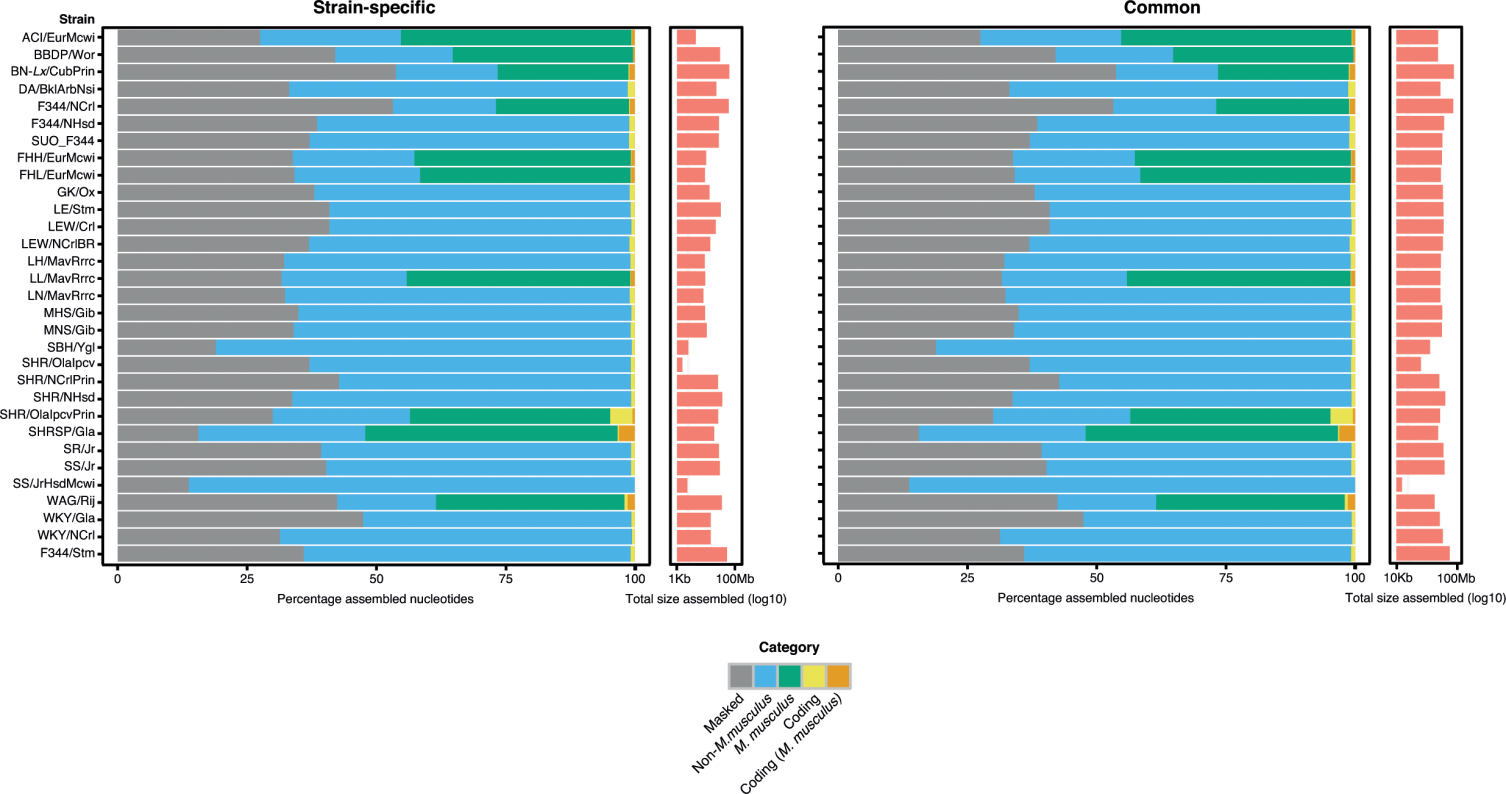
Annotation of strain-specific and common contigs. Contigs that overlap with RepeatMasker in gray, Contig alignment to mouse assembly GRCm38 blue = negative, green = positive, and contigs containing predicted coding sequences in yellow and orange for both protein coding and mouse aligned contigs.

On average 3.36Mb of assembled sequence was classified as strain-specific (30,432 contigs >1Kb and 51 contigs >10Kb). The average size of common assemblies was 4.1Mb (85,741 contigs >1Kb and 104 contigs >10Kb) (Fig 5). Contigs of sufficient length, ≥ 500bp, were used for further analysis and have been made available through the European Nucleotide Archive under accession PRJEB12009.

Both common and strain-specific assemblies contained between 20 and 40% repeat sequences, with large contributions form LINE and SINE elements. These elements have been described to be an important source of intra-species divergence [35,36]. Comparison of the contigs to the mouse genome GRCm38, the closest species to rat with a high quality reference sequence, revealed a slightly larger percentage homology to mouse in strain-specific compared to common sequences (26.99Mb (3.9%) strain-specific vs. 61.53Mb (4.2%), common). Finding more mouse-homologous sequences within strains compared to across strains suggests strain-specific deletions of ancient sequences (i.e. from the most recent common ancestor of rat and mouse).

We used various *ab initio* prediction tools to identify putative coding sequences in the assembled contigs. Putative Open Reading Frames (ORFs) were found in 1,589 strain-specific contigs (~1.92Mb) and in 1,726 common contigs (~2.83Mb) (Fig 5). OrthoMCL identified 1,270 (79.9%) strain-specific and 1,290 (74.7%) common putative ORFs as orthologs of known proteins.

The orthologs of both the strain-specific and common contigs were predominately found in mouse, rat and other rodents: these contigs potentially include (pseudo) genes and gene duplications. We find that mouse-homologous contigs contain the least amount of repetitive elements (20%). Contigs without mouse-homology (non-mouse contigs) contain 40% repetitive elements, suggesting that these could have been introduced through viral retro-transposition or genomic instability in between repetitive regions. Paired-end information was used to identify the genomic location of the contigs, resulting in 70.3% of the contigs being linked to multiple repeat-regions in RGSC5.0. Indicating that there are small (<1Mb) sequences, of possible biological relevance, interspersed within repeat-regions.

To assess the function of the ORFs, we used the Gene Ontology terms of their closest orthologs and performed a gene set enrichment analysis using the Panther 9 algorithm [40]. We found that the biological processes of oxidative phosphorylation and metabolic processes were significantly overrepresented in the data compared to the rat reference (P<0.05). These processes have been shown to have a high amount of redundancy and plasticity [41,42], which could explain their abundance in strain-specific and evolutionary dynamic regions. A doubling of the expected amount of proteins with the molecular function of RNA-directed DNA polymerase activity was also found (Figure C in S1 File). This, in combination with the significant increase in reverse transcriptases (P<0.01), is strongly indicative of an active role of retroviral elements in laboratory rat strain evolution [43].

### Protein structure comparison: *Larp1b* and *Klb*

We analyzed the predicted secondary structure of 2 randomly chosen *in silico*-translated contigs, one strain-specific and one common, with a length close (±1SD) to the mean length. The strain-specific contig S14218 of F344/NHsd showed a 98% similarity with mRNA of rat *La ribonucleoprotein domain family*, *member 1B* (*Larp1b*). The first 9 exons of the *Larp1b* gene are found adjacent to each other in the contig, while exon 10 is missing. Secondary Structure Prediction (SSP) shows strong similarity in all predicted secondary structures, except for the C-terminal region (Fig 5). The last 228bp of the contig were found to be DNA of the interspersed repeat class. Finding a genomic contig with a high similarity to mRNA, in combination with the non-LTR retrotransposon evidence, strongly suggests a pseudogene (instead of duplication in this strain or deletion in other strains): an insertion of host *Larp1b*-cDNA into the genome of the sequenced F344/NHsd sample.

A common contig identified in five substrains (WAG/Rij, SHR/NCrlPrin, WKY/NCrl, SHR/OlalPcv and F344/Nhsd) harbors a spliced incomplete and/or altered klotho-beta (Klb) homolog based on the overlap of common contig-SSP and Klb from *M*. *musculus*. Liver transcriptome data of SHR/OlalPcv indicates that parts of this ORF are expressed (Figure E in S1 File). Previous work in mice showed the correlation between *Klb*-deficiency and chronic renal failure, ageing and altered plasma Ca2+ -levels [44]. Interestingly, four of the sub-strains containing this contig have been phenotyped [45] and show significantly lower levels of plasma Ca2+ compared to available data of other strains similar to our dataset (P<0.05). Our finding of the altered *Klb* and the previous studies suggest that this alternative *Klb* may influence the cellular calcium homeostasis and other functions of the klotho-family (e.g. endocrine factor and co-receptor of Fgf23) in the identified sub-strains but this association clearly warrants further research.

### *In vitro* validation

**Fig 6:**
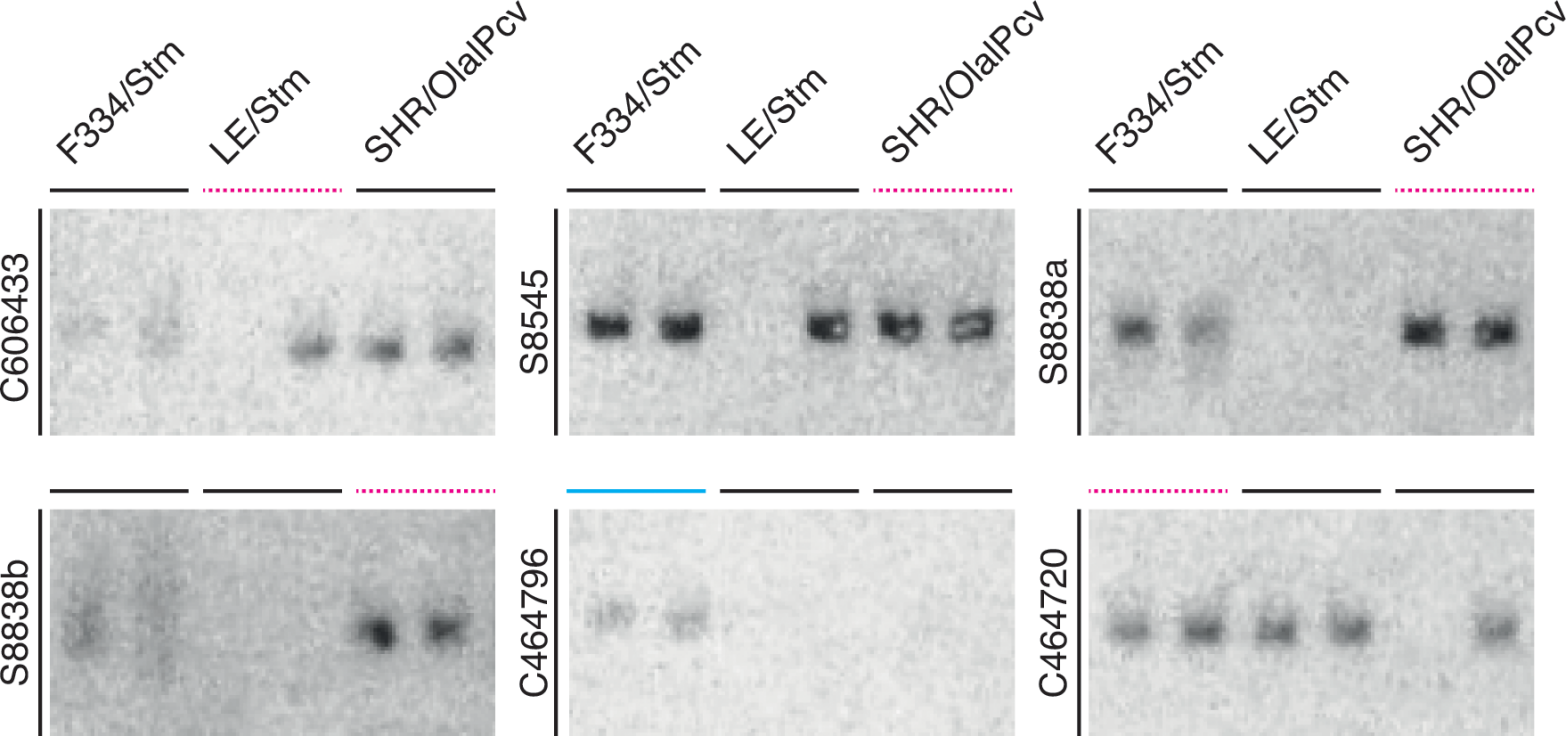
*In vitro* validations; PCR-primers (in duplo) for 6 contigs in 3 strains. Colored bars denote the strain in which the contig was identified; line color and type indicate contig classification; strain-specific (solid cyan) or common (dashed magenta).

We performed a small-scale PCR-based experiment on 6 contigs to assess both the quality of assembly and the assignment of strain-specificity (Fig 6). All 6 contigs were confirmed in the strain it was identified in, showing that our assembly-methods were correct. The assignment of specificity was correct for the assessed strains, the 5 common contigs were found in more than one strain and the strain-specific contig was only found in its own strain.

## Discussion

In our quest to elucidate the origins of unmapped read-pairs of 33 rat-strains, we found sources related to both the wet- and dry-lab procedures. Most importantly, extensive strain-specific genomic segments were found, including regions with potential biological functionality.

The amount of low-quality and short-length reads is less than 50% of all unmapped reads for all strains. This is in line with previous work that showed that less than half of unmapped reads are sequencing artifacts [12]. Since base-call quality score is based on a prediction of the probability of an error at a particular base [46] and such predictions are typically on the safe side, a small amount of low quality reads could have been filtered out incorrectly. Lower quality scores have also been correlated to difficult sequences, including reads with very high and low GC-content and simple sequences (e.g. mononucleotide repeats). Stringent filtering for quality would lead to lower sequence coverage, which may be particularly problematic in regions that have a high GC-content, such as known regions with specific regulatory functions [47,48].

By comparing the current rat reference genome with WGS data obtained from the same animal that was used for creating this reference, we found that 39% of the total unmapped reads are due to missing sequences in the reference genome. As such the use of a single-strain reference pipeline (with a separate strain used for the Y-chromosome) is questionable. We identified regions missing in the reference genome that are common in rat strains as well as regions that are strain-specific. Population specific loci are also found in other genomes: for example the 17q21.31 region in the human genome has a megabase-long population-specific inverted haplo-block and as such has an alternative locus (path) in the reference genome assembly [49,50]. Future work should be focused on creating population-specific alternative paths in reference genomes and a low-memory reference-guided assembly pipeline, which would be more resilient towards population-specific genomic inserts and/or deletions. Recent results and analysis show that the use of alternative paths can lead to more complete analyses and are less sensitive to missing sequences in the reference genome [51].

High amounts of bacterial and viral contaminant-derived reads were identified in several samples. While we focused on unmapped reads, more in-depth research of contaminants could investigate the amount of contaminant-derived mapped reads which lead to falsely mapped reads in the reference, similar to a recent study in humans [19]. Another source of contamination is inherent to the sequencing methods used: adapters and positive controls. Of the contamination, a large part can be attributed to the positive control sample (phiX) of the Illumina platform. Sequencing adapters are found in this study to lead to high amounts of (unnecessary) unmapped reads, as these adapters lead to mismatches during mapping. A pre-mapping filtering script is generally not used, since such a step is computationally heavy and unmapped reads are not used in analyses. However, we show that this could lead to a potentially large amount of contaminant-derived reads.

The distribution of ‘unmapped reads’ across strains follows the (polymorphism-based) phylogeny, while this could be expected it was never quantified in an unbiased way. Previous studies have shown that there is similarity between contigs of unmapped reads in mice-strains and that there is a biotype-based clustering in aphid unmapped reads [12,15]. Our study shows that there is up to 90% similarity between unmapped reads of samples with a shared ancestor. Even though we filter against Y-chromosomal BACs a large portion of Y-chromosomal contigs remains in the data, as strains cluster firstly on gender, rather than phylogeny. The incomplete removal of this signal using BACs is likely due to the high variability of the Y-chromosome between strains: the strain (SHR/Akr), used for the Y-chromosomal BAC-contigs, has a small Y-chromosome compared to BN, which ranks amongst the largest [37].

When comparing the total size of strain-specific genomic segments per sample in our study with a previous study in mice [21], we find similar amounts of strain-specific genomic segments in inbred rat strains. The effect on sequencing depth and library size on *de novo*-results is also apparent: F344/Stm (mate-pair), F344/NHsd (35.4x) and DA/BklArbNsi (32.6x) have an approximately tree-fold higher amount of strain-specific bases in their assemblies. Interestingly, there appears to be a trend between higher amounts of total sequencing reads (e.g. DA/BklArbNsi, F344/NHsd and LE/Stm) with respect to the amount of repeats in their respective contigs. This could be due to a higher amount of retroviral activity, but is more likely to be the results of higher coverage resulting in better *de novo* assemblies.

The finding of putative protein-coding regions in the strain-specific contigs and the high percentage of interspersed elements is in line with the previous studies on (non-)LTR retro-transposons and the evolution of the genome in mammals [19,20]. Due to the limited size of the contigs, we do not always have sequence extending up to the poly-A tails and/or promotors, which prevents the distinction between functional (i.e. duplicated) genes or pseudogenes.

**Fig 7:**
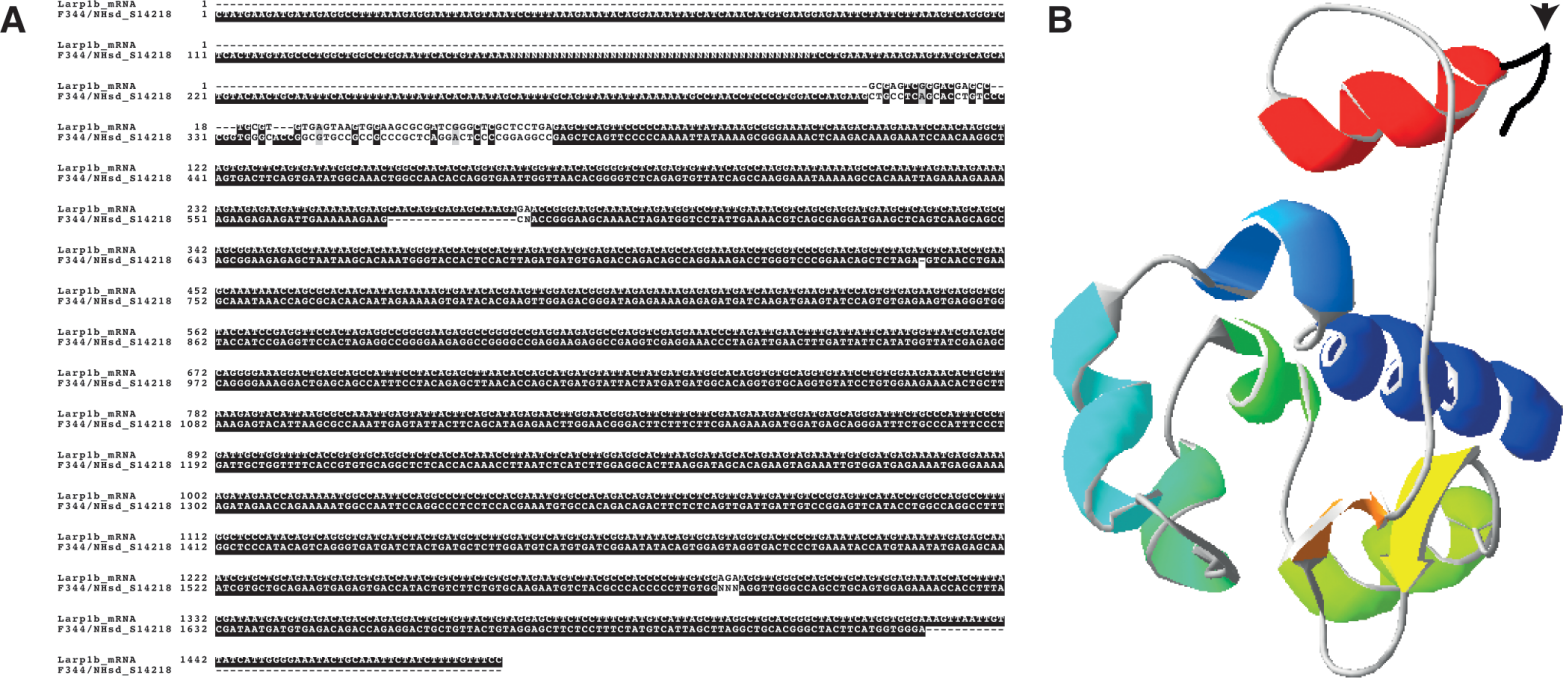
***Larpb1* in F344/NHsd A)** Alignment of F344/NHsd contig S14218 with rat *Larp1b*-mRNA shows strong similarity with the first nine (of ten) exons. B) Superimposed SSP of F344/NHsd contig S14218 on SSP of *Larp1b* mRNA. Only the N-terminal structure, in black, -denoted with the arrow- is missing from S14218.

The identification of the (pseudo)gene of *Larp1b* is a good example of the potential impact of an assembly based approach for unmapped-reads. The contig contained a sequence highly similar to the mRNA of *Larp1b* with a bordering SINE-sequence, it has a near-complete ORF without introns (the predicted secondary protein structure is similar, Fig 7). The altered *Klb* in strains with lower amounts of cellular calcium homeostasis is an example of ability of our analysis to find a potential causal relationship based on matching strain distribution patterns of genotypes and phenotypes.

## Conclusions

This study shows that a large portion of unmapped reads in the rat model system are of biological interest, rather than sequencing-artifacts. This is largely due to the use of an incomplete reference genome, derived from a single substrain, which is a problem for all publically available genomes apart from human. The large amounts of biologically relevant data in the unmapped reads, as demonstrated by their potential to reconstruct the phylogentic tree, highlights the amount of sequences missing in the reference-strain, indicating that all re-sequencing experiments should account for the shortcomings of using a single reference genome. Using multi-path genome assemblies will be a partial solution to this but it fails to account for recently acquired material. The efforts described here can contribute to the identification and creation of alternative paths, while also identifying novel sequences in a systematic way. Until alternative paths are systematically included and part of analysis procedures, researchers should be aware of the missing sequences or use approaches such as described here to detect these. Rat based studies can use our resource for mapping and in-depth analysis of strain-specific genotype-phenotype relationships. Analysis of the strain-specific genomic segments resulted in the discovery of candidate coding segments and homologous sequences, which both have the potential for playing a role in the strain-phenotypes.

## Methods

For this study, paired-end Illumina HiSeq WGS-data of 30 rat strains was used [17,24,26]. An additional Strain of Unknown Origin (SUO) was added to the dataset [17]. SHR/OlalPcv paired-end WGS-data (Illumina GAII-platform) and the mate-pair data of F344/Stm was also included, which brought the total amount of strains to 33 [24,52] (Table 1). The raw data was mapped against the Brown Norwegian (BN/SsNHsdMCW) reference genome, version 5.0 (RGSC5.0) with BWA mem (0.7.5a-r405), base quality scores were recalibrated with the genome analysis toolkit 3.1–1 [53] and PCR duplicates were removed with Picard tools MarkDuplicates 1.118 [54]. Unmapped pairs were extracted with SAMtools 0.1.14 [55]. Pairs with only one mapped mate were also extracted: these were mapped with the anchored split-read mapper Pindel 0.2.5a1 [56]. Correctly mapped pairs from Pindel (originating from deletions, short insertions, inversions or tandem duplications) are considered to be unmapped due to genomic variation (Fig 1A). A full description of the performed analysis, utilized third-party software and utilized custom scripts are available in the “GitHub” repository, http://git.io/scrapheap.

### Quality control

The Illumina TruSeq-2/3 adapters were clipped from the unmapped pairs, including the unmapped pairs of the Pindel-mapping, using Trimmomatic 0.30 [4]. Base-quality clipping was done by using a 25nt sliding window with a phred33-score quality threshold of 25. The pairs were then filtered on read-length, where both reads must be longer than fifty percent of the expected size. Pairs that did not meet these criteria were considered to be sequencing artifacts and/or errors.

### Mapping-based filtering

The remaining read-pairs were compared with the alternative reference genome, Y-chromosomal BACs and a contamination databases with an in-house pipeline (available on request). This pipeline maps read-pairs to all three databases with BWAmem (0.7.5a-r405) and extracts pairs that do not map in-pair at the correct insert-size against any of the databases. Furthermore, it generates hit-counts of all three databases, including overlapping read-pairs (e.g. read-pairs that map to both Celera and Y) (Fig 1B).

The alternative reference for *R*. *norvegicus* from Celera Genomics, Rn_Celera, is composed of 29 million BN-fragments and 8 million SD-fragments [57]. SOLiD WGS BN/SsNHsdMCW-reads were mapped against Rn_Celera and the mapped regions were selected [31]. This gives the possibility to differentiate between read-pairs mapping to true SD-specific regions or to missing regions in RGSC5.0. This, because Celera-regions mapped by BN/SsNHsdMCW SOLiD-reads are considered to be regions that should also be in RGSC5.0, which is an BN/SsNHsdMCW-assembly.

Data from the *Rattus norvegicus* Chromosome Y Mapping Project, containing BAC-contigs from Solexa- and Sanger-sequenced SHR/Akr, is used for finding Y-chromosomal read-pairs [58]. Reads from repeats and from cross-genome homologous regions could also map to these BAC-contigs: this would lead to high numbers of false positives (e.g. the identification of Y-chromosomal read-pairs from (fe)male samples). To investigate this, male and female paired-end WGS-data was aligned to the BAC-contigs with BWAmem, extracting all properly mapped pairs [59]. In mice, 50–66% of the Y-chromosome synapses with the X-chromosome [33]. If the BAC-contigs are complete and a correct representations of the rat Y-chromosome and these putative percentages of pseudoautosomal regions (PAR) in mice are similar in rat, similar percentages mapped read-pairs of the female compared to the male data should be found (Supplemental text 1). For the scope of this study, however, all mapped read-pairs to the (putative PAR-regions of) BAC-contigs were removed from the dataset: they were considered to not be strain-specific, as they map to (the Y-chromosome of) SHR/Akr.

The prokaryotic, viral and fungal RefSeq-genomes (V.61) were used for the contaminant-database. Contamination is defined here as non-*animalia* and can be the result of (in)direct contamination of a sample or from a positive control in the sequencer, like the bacteriophage PhiX174 of the Illumina HiSeq-platform. In order to keep the amount of false positives (i.e. reads mapping to genomes of closely-related species to rat) low, we did not include *animalia*-genomes in this step. If contamination of *animalia* was present in the dataset, we were able to identify this in the OtherMCL-analysis. To produce an overview of the contaminating bacteria, mapped regions in the bacterial genomes were pooled at the genera-level. This was found to be the deepest taxonomic layer possible for the resolution of 500bp, the insert size of the read-pairs, due to the high percentages of whole genome sequence identity within bacterial genera [60].

### Comparison of relevant reads

Compareads 2.0.2 [61] was used for the comparison of the remaining read-pairs of different Illumina paired-end sequenced strains to each other. GK/Ox, SBN/Ygl, WKY/NHsd and WAG/Rij were discarded for this analysis. This was done because the median read-length was too low (50nt), low amounts of read-pairs (<30k) and/or because of GC-bias. Since read-pair filtering was already done, only the direct comparison- and extraction-scripts of Compareads were used. For the comparison, two K-mers of 40% read-length were required to be considered similar.

### *De novo* assembly pipeline

For the assembly of the remaining read-pairs, a SOAPdenovo-based pipeline was used on a 48-core Linux cluster with 500GB RAM. Pre-assembly optimization was done with SOAPEc v2.01 in HA-mode, which corrects sequencing-errors based on low-frequency Kmers [62]: because the read-pairs were already filtered on base-quality, the phred-quality threshold was lowered to 30. Next, the optimal Kmer for *de novo* assembly was found using Kmergenie v1.5854 [63], which uses K-mer frequency-distribution estimation for finding the Kmer with the maximum amount of unique Kmers, leading to more accurate *de novo* assembly-inputs.

SOAPdenovo v2.04 was used as the primary assembler, due to the ability to handle large genomes and 100-bp Illumina paired-end sequences, while keeping the computational burden low. This assembler was also used in the *de novo* assembler-comparison Assemblathon 2 for a similar dataset [64]. Because the found optimal Kmers were never higher than 59, the 63mer-version was used. Post-assembly optimization, filling gaps in scaffolds and error-correction of contigs derived from reads with incorrect insert-sizes in the contigs, was done with the GapCloser tool, which was specifically designed for downstream analysis of SOAPdenovo results. A distance-plot was made by aligning all strain-assemblies to each other with BLAT version 35x1 [65] and calculating the distance score as described in [66].

### Strain-specific and common contigs

The assembled sequences of each substrain were aligned to all other (sub)strain assemblies with BLAT version 35x1 [65]. Substrains were considered to be from the same strain if they a) have the same strain-name (i.e. the name in front of the slash) and/or b) have a genetic distance of less than 0.02 in the paper of [26]. A Python-script (available on http://git.io/scrapheap) filtered out contigs longer than 500bp, have a matching sequence of more than 100bp and an overall similarity of more than 80%. Common contigs were clustered with CD-HIT version 4.5.4 [67] and clusters of more than 2 sequences were aligned with Clustal Omega version 1.2 [68].

### Sequence prediction and validation

Analysis of repetitive elements in the contigs was performed with RepeatMasker 4.0.3, using the Repbase-derived *R*. *norvegicus* library version 20140131 [69,70]. Augustus 3.0.1 [71] was used for *ab initio* gene prediction of the non-clustering contigs, using additional rat EST-data from UniGene as extrinsic information. The resulting peptides were then assigned to orthologous groups using OrthoMCL 5 [72]. Using the hypothesis that the chance of finding *de novo* proteins is magnitudes lower than finding orthologous proteins, we only keep the predicted protein with an assigned orthologous group. For the analysis of the examples, we also used SWISS-MODEL for automated protein structure modeling [73]. Strain-comparison statistics were calculated with the Welch T-test.

### *In vitro* validation

To assess both the quality of assembly and the assignment of strain-specificity, we performed a small-scale experiment. Primer-sets were designed for contigs of three different sub-strains and PCR was performed on samples of each strain. Primer sets form common contigs should lead to amplification of the sequence in the strain it was identified in as well as in other strains. Strain-specific contigs should yield primers that only lead to amplification in samples belonging to that specific strain.

### Consent

Not applicable

## Supporting Information

S1 File Supplementary file 1 containing all supplementary figures. (PDF)
