# Supplementary material for: The Genomic Scrapheap Challenge; Extracting Relevant Data from Unmapped Whole Genome Sequencing Reads, Including Strain Specific Genomic Segments, in Rats
Source: PLoS One. 2016 Aug 8;11(8):e0160036. doi: 10.1371/journal.pone.0160036 (PMC4976967; doi:10.1371/journal.pone.0160036)
Supplement: S1 File — (PDF) [file pone.0160036.s001.pdf]

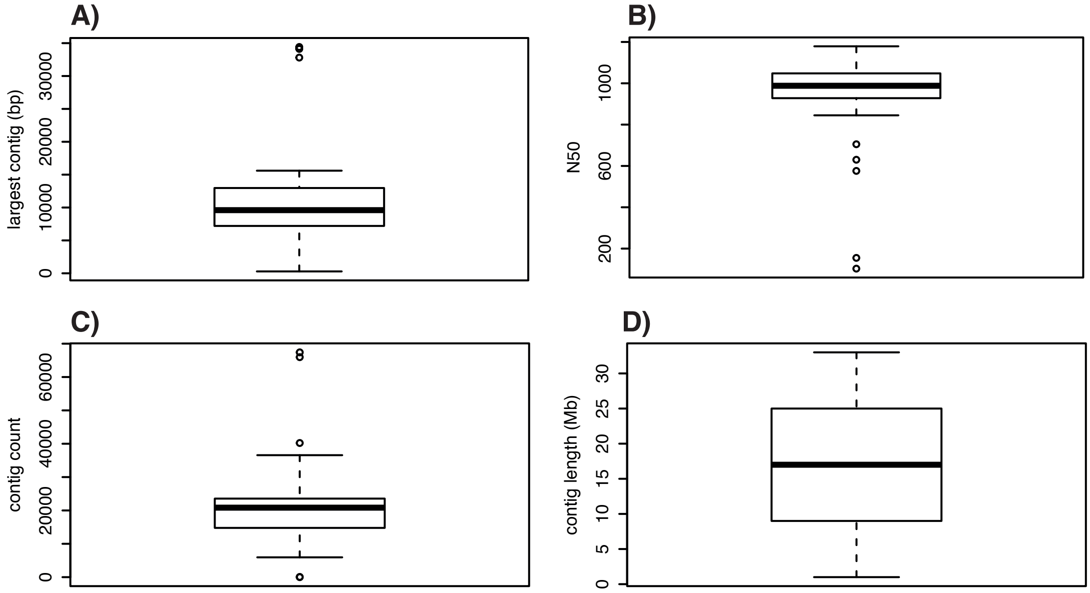

Supplementary figure A - *De novo* assembly metrics. Per-(sub)strain distribution of **A)** largest contig size, **B)** N50, **C)** amount of contigs and **D)** total contigs length.

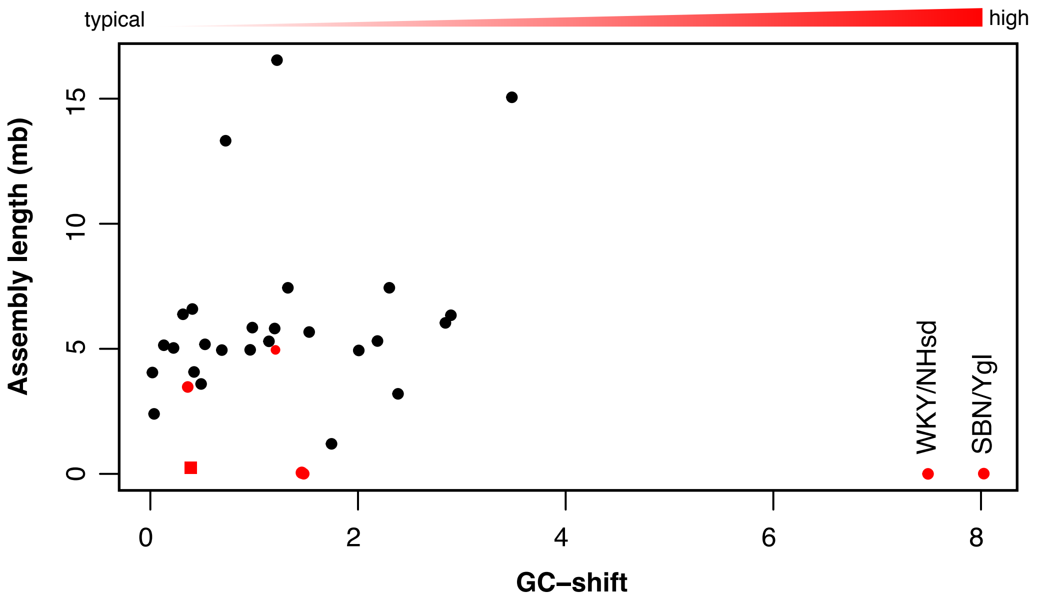

Supplementary figure B - The effects of GC-shift and low coverage on the results of novo assembly. Red dots denote the low (<20X) coverage strains: BBDP/Wor, LEW/NCrlBR, SR/Jr, SS/Jr WKY/NHsd and SBN/Ygl. The blue dot is sequenced on Illumina GAII (SHR/Olalpcv).

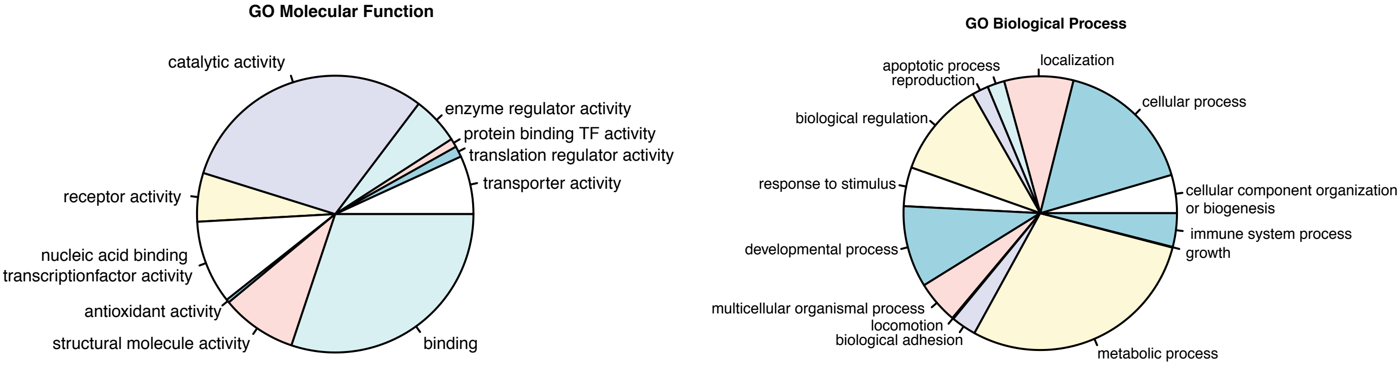

Supplementary figure C - Functional classification of putative ORFs. Panther 9 output of the putative ORFs: the molecular functions “catalytic activity” and “binding” contribute to more than 50% of the found GO-terms. Metabolic processes are the largest biological process in the GO-terms.

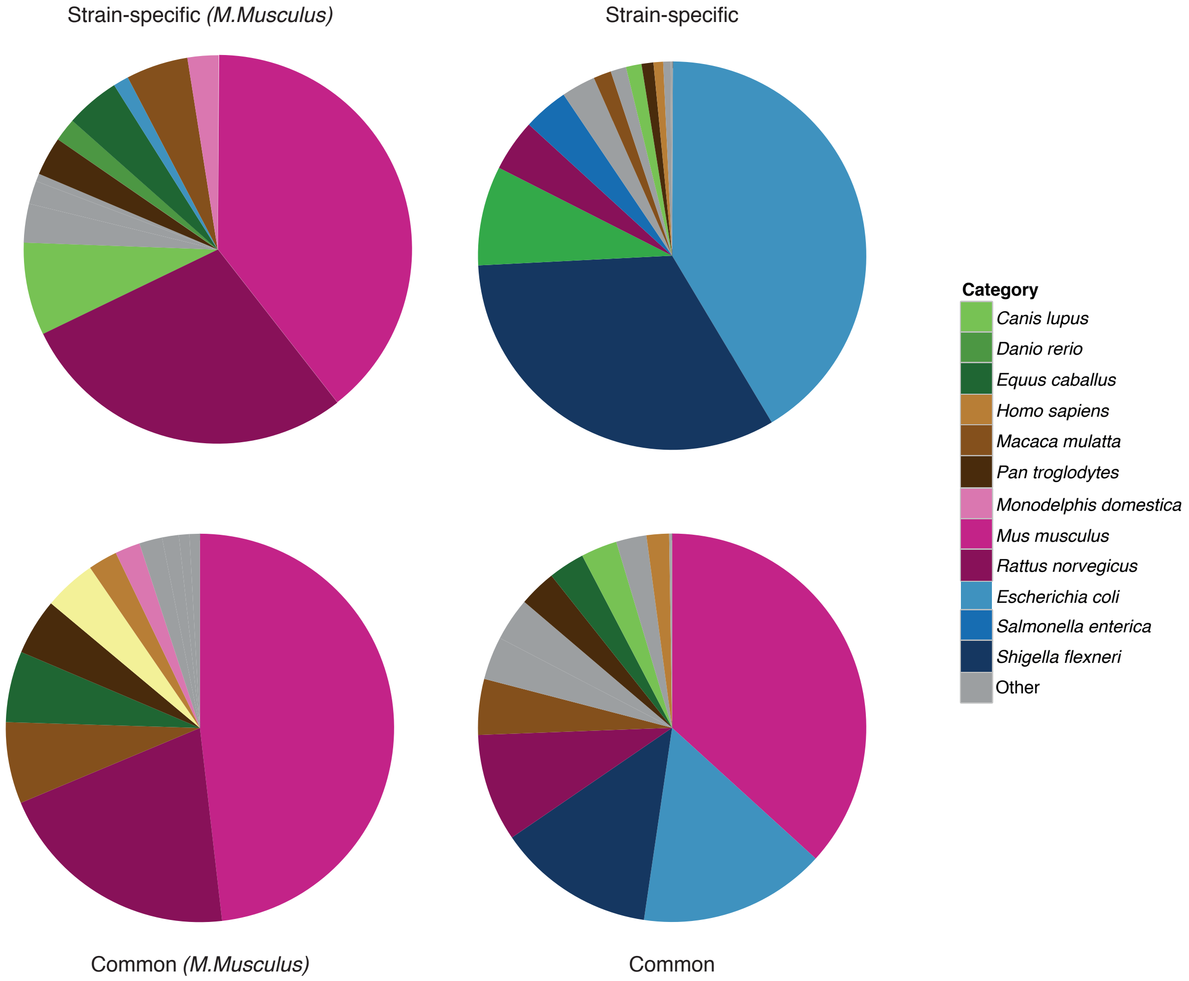

Supplementary figure D - Organism-distribution of orthologs. Based on the found orthologs of OrthoMCL, the majority of found ortologs is found in rodents.

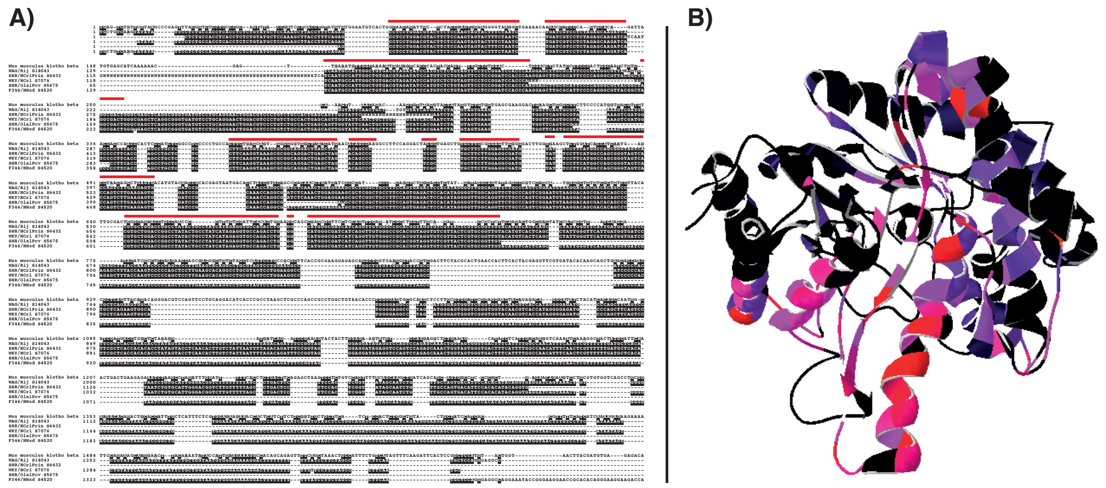

Supplementary figure E - Klotho beta (KLB). Large amounts of overlap are visible in both the DNA- and Protein-alignment **A)** Alignment of *Mus musculus* KLB-mRNA and the countigs of WAG/Rij, SHR/NCrlPrin,WKY/NCrl,SHR/OlaIP-cv and F344/Nhsd. The red line denotes coverage (>10X) of SHR/OlaIPcv-liver RNA-seq data. **B)** Predicted proteins, superimposed on mouse KLB structure prediction: Magenta colouring shows areas with high amounts of overlap.
